# Efficacy of Electrochemotherapy in Breast Cancer Patients of Different Receptor Status: The INSPECT Experience

**DOI:** 10.3390/cancers15123116

**Published:** 2023-06-08

**Authors:** Claudia Di Prata, Matteo Mascherini, Alastair MacKenzie Ross, Barbara Silvestri, Erika Kis, Joy Odili, Tommaso Fabrizio, Rowan Pritchard Jones, Christian Kunte, Antonio Orlando, James Clover, Siva Kumar, Francesco Russano, Paolo Matteucci, Tobian Muir, Francesca de Terlizzi, Julie Gehl, Eva-Maria Grischke

**Affiliations:** 1 Department of Surgery, Oncology and Gastroenterology (DISCOG), University of Padua, 35128 Padua, Italy; 2 Department of Surgery, IRCCS Ospedale Policlinico San Martino, 16132 Genoa, Italy; mascherinimatteo@gmail.com; 3 Department of Plastic and Reconstructive Surgery, St Thomas’ Hospital, London SE1 7EH, UK; 4 Oncology and Haematology Unit, Azienda Unità Sanitaria Locale Socio Sanitaria (AULSS) 3 Serenissima-Mirano, 30035 Venice, Italy; barbara.silvestri@aulss3.veneto.it; 5 Department of Dermatology and Allergology, University of Szeged, H-6720 Szeged, Hungary; ksgbrll@gmail.com; 6 Department of Plastic Surgery, St. Georges University Hospitals NHS Trust, London SW17 0QT, UK; joy.odili@stgeorges.nhs.uk; 7 Unit of Plastic Surgery, Centro di Riferimento Oncologico della Basilicata (IRCCS-CROB), 85028 Rionero in Vulture, Italy; tommaso.fabrizio@crob.it; 8 Department of Plastic Surgery, Edge Hill University, Ormskirk L39 4QP, UK; rowan.pritchardjones@sthk.nhs.uk; 9 Department of Plastic Surgery, University of Liverpool, Liverpool L7 8TX, UK; 10 Abteilung für Dermatochirurgie und Dermatologie, Artemed Fachklinik München, 80336 Munich, Germany; christian.kunte@artemed.de; 11 Department of Plastic and Reconstructive Surgery, Southmead Hospital, North Bristol NHS Trust, Bristol BS10 5NB, UK; antonio.orlando@nbt.nhs.uk; 12 Department of Plastic Surgery, Cork University Hospital, T12 DC4A Cork, Ireland; j.clover@ucc.ie; 13 Cancer Reseach@UCC, University College Cork, T12 YN60 Cork, Ireland; 14 Department of Plastic Surgery, Queen Victoria Hospital National Health Service (NHS) Foundation Trust, East Grinstead RH19 3DZ, UK; siva.kumar1@nhs.net; 15 Soft-Tissue, Peritoneum and Melanoma Surgical Oncology Unit, Veneto Institute of Oncology IOV—IRCCS, 35128 Padua, Italy; francesco.russano@iov.veneto.it; 16 Hull University Teaching Hospitals NHS Trust, Hull HU3 2JZ, UK; p.matteucci@nhs.net; 17 Department of Plastic Surgery, James Cook University Hospital, Middlesbrough TS4 3BW, UK; tobian.muir@nhs.net; 18 IGEA S.p.A. Biophysics Department, 41012 Carpi, Italy; f.deterlizzi@igeamedical.com; 19 Center for Experimental Drug and Gene Electrotransfer (C*EDGE), Department of Clinical Oncology and Palliative Care, Zealand University Hospital, 4000 Roskilde, Denmark; kgeh@regionsjaelland.dk; 20 Department of Clinical Medicine, Faculty of Health and Medical Sciences, University of Copenhagen, 2200 Copenhagen, Denmark; 21 Department of Gynecology, University Hospital of Tübingen, 72076 Tübingen, Germany; eva-maria.grischke@med.uni-tuebingen.de

**Keywords:** electrochemotherapy, breast cancer, cutaneous metastases, receptor status

## Abstract

**Simple Summary:**

Electrochemotherapy has proven to be an efficient treatment for cutaneous metastases of various cancers including breast cancer (BC). The large number of patients collected within the INSPECT database provides the possibility of a differentiated analysis on BC with different receptor statuses (estrogen receptor and HER2 receptor). Patients with BC presenting cutaneous metastases were retrieved from the INSPECT database and divided by their receptor status: HER2+, HR+ (ER/PgR+), and TN (triple negative). ECT treatment is equally effective among groups, despite different conditions, age, time since diagnosis, previous or concomitant treatments, and treatment characteristics. Response and local tumor control seem to be better in multiple small lesions than in big armor-like lesions, suggesting that treating smaller, even multiple, lesions at the time of occurrence is more effective than treating bigger long-lasting armor-like cutaneous lesions.

**Abstract:**

Electrochemotherapy has been proven to be an efficient treatment for cutaneous metastases of various cancers. Data on breast cancer (BC) patients with cutaneous metastases were retrieved from the INSPECT database. Patients were divided by their receptor status: HER2+, HR+ (ER/PgR+), and TN (triple negative). Groups were similar for histological subtype and location of the nodules. Most patients were previously treated with surgery/systemic therapy/radiotherapy. We found no differences in the three groups in terms of response ratio (OR per patient 86% HER2+, 80% HR+, 76% TN, *p* = 0.8664). The only factor positively affecting the complete response rate in all groups was small tumor size (<3 cm, *p* = 0.0105, *p* = 0.0001, *p* = 0.0266, respectively). Local progression-free survival was positively impacted by the achievement of complete response in HER2+ (*p* = 0.0297) and HR+ (*p* = 0.0094), while overall survival was affected by time to local progression in all groups (*p* = 0.0065 in HER2+, *p* < 0.0001 in HR+, *p* = 0.0363 in TN). ECT treatment is equally effective among groups, despite different receptor status. Response and local tumor control seem to be better in multiple small lesions than in big armor-like lesions, suggesting that treating smaller, even multiple, lesions at the time of occurrence is more effective than treating bigger long-lasting armor-like cutaneous lesions.

## 1. Introduction

Breast cancer is one of the most frequent cancers worldwide. Data from the 2019 US SEER (Surveillance, Epidemiology and End Result program) registry point out that the incidence of breast cancer among the US population is almost 134 new cases per 100.00 inhabitants per year, with an increasing trend and the prevalence of the female population affected is 2.3% of the total population [1]. Breast cancer is also one of the most frequent causes of cutaneous and subcutaneous metastases [2].

Clinically, skin and subcutaneous metastases could present themselves in different ways: they could be single or multiple, could be asymptomatic or could bleed, ulcerate, be painful, and lead to infections.

Treatment of these lesions varies accordingly. Surgery could be a first option in single small metastasis, while treatment of larger or multiple lesions spreading into a larger area could be challenging. Systemic therapies could be proposed according to tumor subtype (histology, immunohistochemistry, hormonal status, receptor status), while radiotherapy could be chosen if not previously delivered [3,4].

Electrochemotherapy is recognized as a safe and valid treatment option to manage these lesions; it could be performed in previously irradiated areas and could be repeated multiple times [5,6,7,8,9]. Various mono- and multicentric studies demonstrated high response rates in terms of local control, with the overall response rate up to about 80–90% and complete control rate up to about 60% [7,8,10].

It is also demonstrated in different cancer settings (e.g., melanoma) that ECT could be safely performed concurrently to other systemic treatments, leading to a cutaneous local control that can improve the patient’s quality of life and also have a benefit for their psychological well-being [11,12,13]. Moreover, some studies suggest that the combination of ECT with other systemic therapies could be beneficial, and this interaction is being explored [14,15]

Predictive factors to ECT response in breast cancer cutaneous and subcutaneous metastases have been previously investigated in small studies and some have been reported (e.g., small tumor size, absence of visceral metastases, estrogen receptor positivity, low Ki-67 index, lower body mass index, reduced body surface, absence of previous radiation treatment, concurrent systemic therapies) [7,8,10,16].

We present here the analyses from the INSPECT network on 171 patients treated with ECT for cutaneous and subcutaneous metastases from breast cancer from January 2010 to November 2020. Patients were divided into three groups regarding hormonal and immunohistochemical status: HER2+, HR+ (ER/PgR+), and TN (triple negative). The primary aim of the study was to evaluate any differences in terms of ECT response and to predict different factors to the treatment’s response, if any, related to hormonal status. The secondary goal was to evaluate survival (local progression-free survival, overall survival).

## 2. Materials and Methods

### 2.1. Patients

Patients were recruited and treated at institutions in the INSPECT network. Centers uploaded patient data prospectively in the International Network for Sharing Practices of ECT (InspECT) register (http://www.insp-ect.org). Approval from the ethics committee and data protection authority was according to guidelines of each institution and to the rules of Good Clinical Practice (Declaration of Helsinki).

Patients eligible for inclusion had histologically proven breast cancer with measurable cutaneous or subcutaneous metastases suitable for application of electric pulses. Patient selection was based on institutional preferences, including referral after multi-disciplinary discussion for patients with symptomatic cutaneous metastasis when other treatment modalities failed or were not possible. They were offered standard treatment options when possible, were 18 years old or older, had an Eastern Cooperative Oncology Group performance status ≤2, had life expectancy of at least 3 months and, where appropriate, were using adequate contraception. Patients were ineligible if they previously had allergic reactions to bleomycin or to any of the components required for anesthesia, if the cumulative dose of 250 mg (400,000 IU) bleomycin/m^2^ had previously been exceeded, and in case of chronic renal dysfunction (serum creatinine >150 mmol/L) or acute lung infection. Clinical information retrieved by the database included: demographic characteristics, number of treated lesions, site and size of the largest lesion, previous irradiation, and duration of follow-up.

Data on histology, hormone receptor status, HER2 receptor status, and previous treatments were also collected. Patients under concomitant systemic treatment or who started new systemic antineoplastic treatment after electrochemotherapy were included in the analysis.

### 2.2. Procedure

ECT was delivered based on European Standard Operating Procedures for Electrochemotherapy (ESOPE) updated guidelines [9]. Bleomycin was administrated in one of the following ways: intratumorally at a dose of 1000 IU/cm^3^ for tumor volume < 0.5 cm^3^, 500 IU/cm^3^ for tumor volume between 0.5 and 1 cm^3^, 250 IU/cm^3^ for tumor volume > 1 cm^3^, or intravenous at 15,000 IU m^2^ body surface. Electroporation was achieved using the Cliniporator (IGEA, Carpi, Italy), delivering 8 pulses of 100 ms at 1 kV/cm. Electrode choice was guided by the standard operation procedure (SOP) [9,17], which advocates parallel array electrodes for lesions less than 3 cm, with hexagonal array preferred for larger lesions, and subsequent analysis indicated that clinician preference dictates exceptions for this.

After electrochemotherapy, the treated metastases were covered with standard dressings where necessary.

### 2.3. Response Evaluation

Evaluation of the local tumor response was measured via dimensions of the treated lesions. The response was registered for each target lesion at each follow-up visit, and data of response at 1 and 2 months after electrochemotherapy were considered for local tumor response, according to the Modified Response Evaluation Criteria in Solid Tumors (RECIST) [18]: complete response (CR) was defined as disappearance of the target lesion; partial response (PR) with at least 30% decrease in the diameter of the target lesion; progressive disease (PD) with at least 20% increase in the diameter of the target lesion; and stable disease (SD) with neither sufficient shrinkage to qualify for PR nor sufficient increase to qualify for PD. In some cases with ulcerated tumors, evaluation was not possible because of crust formation. Data on local progression-free survival (LPFS) and overall survival (OS) were also collected.

### 2.4. Safety and Toxicity

Safety was reported in the form of adverse events using Common Toxicity Criteria version 5.1. Particular focus was put on local symptoms, such as odor, suppuration, hyperpigmentation, ulceration, and pain. Furthermore, patients were asked if they would potentially agree to another session as a measure of how patients felt about the treatment procedure. Symptomatology assessment was conducted before treatment and each follow-up visit and was specifically analyzed at 3 time points: before treatment, at 1 month from treatment, and at 2 months from treatment. Pain intensity was evaluated using the numeric rating scale (NRS) for pain. The NRS is a unidimensional 11-point numeric scale in which the patient is asked to indicate a whole number between “0” as “no pain” and “10” as “worst pain”.

### 2.5. Statistical Analysis

Descriptive methods were used for statistical analysis using NCSS version 9.16. Continuous variables were described using mean, standard deviation, median value and range, and categorical variables by absolute number and percentage. Comparison among groups was performed via ANOVA test (continuous) and contingency analysis with the χ^2^ test (categorical) for trend.

Univariate analysis was performed in each subgroup using a logistic regression model for complete response, using the investigated variables: oligometastatic disease, previous systemic treatment, concomitant systemic treatment, previous irradiation of treated lesions, lymphoedema, lesions’ size, lesions’ number, electrode type, and current.

Local tumor control was expressed as local progression-free survival, which was the time from electrochemotherapy up to the date of relapse or progression or last follow-up. Survival curves for local progression-free survival (LPFS) and overall survival (OS) were calculated using the Kaplan–Meier model. Cox regression analysis was performed to identify variables affecting LPFS and OS.

Significance of tests was reported with *p*-value, where a value <0.05 was considered as statistically significant.

## 3. Results

### 3.1. Patients

A cohort of 171 patients with a diagnosis of breast cancer and cutaneous or subcutaneous lesions were extracted from the INSPECT database. They were treated with ECT in the period January 2010–November 2020 in 16 European centers (Tuebingen, Padova, London (St. Georges, Guy’s, and St Thomas’ Hospitals) Copenhagen, Middlesbrough, Szeged, Cork, East Grinstead, Munchen, Rionero in Vulture (Potenza), Mirano (Venice), Munchen, Liverpool, Bristol, Castle Hill, Genova).

They were divided into three groups according to their receptor status: HER2+ (all patients with HER2 overexpression), HR+ (patients with either ER or PG expression (normal HER2), and TN (triple negative, without HER2+, ER, or PG expression). Descriptive characteristics of the patients are reported in Table 1.

Almost all patients underwent previous treatments, as reported in Table 2.

The characteristics of the treated lesions and ECT parameters at the ECT session are reported in Table 3.

### 3.2. Toxicity

**Figure 1:**
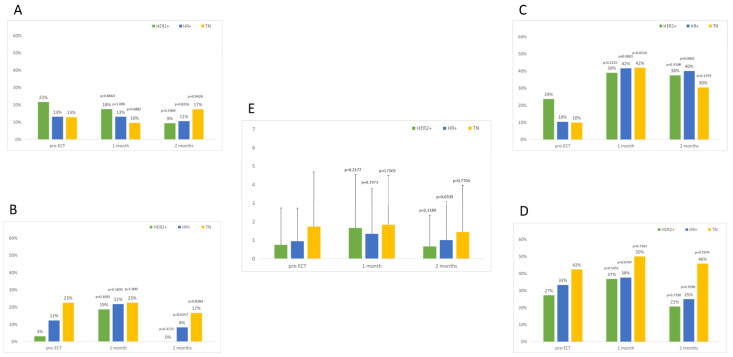
Percentage of patients suffering local symptoms before and after ECT. (**A**) Odor, (**B**) suppuration, (**C**) hyperpigmentation, (**D**) ulceration, (**E**) pain VNS (mean values and standard deviation). *p* values reported are for comparisons within each group with pre-ECT values. Differences among groups are always non-significant.

Local symptoms were mild (grade I/II), and the percentage of patients suffering them showed a similar trend over time in the three groups, as reported in Figure 1. A slight increase in local symptoms can be observed 1 month after ECT, rapidly decreasing to pre-ECT values or even lower values, especially for what concerns odor, suppuration, ulceration, and pain.

### 3.3. Local Response to ECT

Response to ECT was assessed at around 2 months follow-up. Response per patient was: 49% CR, 37% PR, 7% SD, and 7% PD in the HER2+ group; 45% CR, 35% PR, 12% SD, and 9% PD in the HR+ group; 33% CR, 39% PR, 15% SD, and 9% PD in the TN group (*p* = 0.8664). Response per nodule was similar: 52% CR, 37% PR, 4% SD, and 7% PD in the HER2+ group; 55% CR, 31% PR, 10% SD, and 4% PD in the HR+ group; 49% CR, 34% PR, 5% SD, and 11% PD in the TN group (*p* = 0.3846).

**Figure 2:**
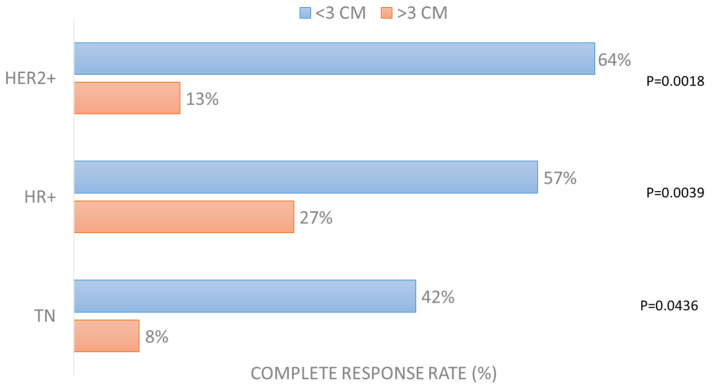
Complete response rate (%) per patient according to largest lesion size (dichotomized at dimeter of 3 cm).

Among all factors considered in the analysis (oligomestastatic condition, previous systemic treatment, concomitant systemic treatment, pre-irradiation of treated lesions, presence of lymphoedema, nodules’ size, nodules’ number, electrode used, current applied), the only significant factor affecting the achievement of complete response per patient was the size of the largest lesion in all three groups (Table S1). The CR rate in the HER2+ group for lesions smaller than 3 cm was 64% vs. 13% in those larger than 3 cm (*p* = 0.0018); in the HR+ group, the CR rate in small lesions was 57% vs. 27% of larger ones (*p* = 0.0039); in the TN group, the CR rate was 42% in smaller lesions and 8% in larger lesions (*p* = 0.0436), as reported in Figure 2.

### 3.4. Local Progression-Free Survival

Patients were followed for different periods, depending on the center and availability of the patients themselves. The mean follow-up time was 12 ± 18 months (median 6.6, range 2–135) and was similar among groups (*p* = 0.0710).

**Figure 3:**
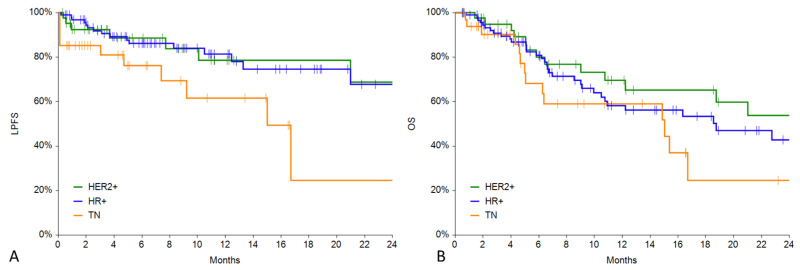
(**A**) LPFS in the 3 groups. (**B**) Overall survival in the 3 groups.

**Figure 4:**
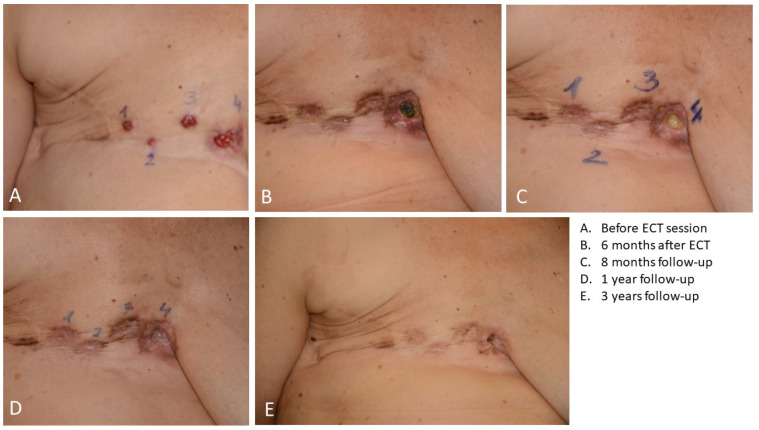
Local progression-free survival up to 3 years in a HER2+ patient treated with ECT.

During follow-up, 28 patients (16%) underwent local progression (12% in HER2+, 18% in HR+, 18% in TN) after a mean period of 13.7 ± 30.7 months. The local progression-free survival curve was significantly lower for the TN group in comparison with the HER2+ (*p* = 0.0390) and HR+ (*p* = 0.0151) groups (see Figure 3A). One-year local progression-free survival was 78% (C.I. 62–95%) in the HER2+ group, 81% (C.I. 72–91%) in the HR+ group, and 61% (C.I. 39–84%) in the TN group. In Figure 4, an example of local progression-free survival up to 3 years in a HER2+ patient is reported.

**Figure 5:**
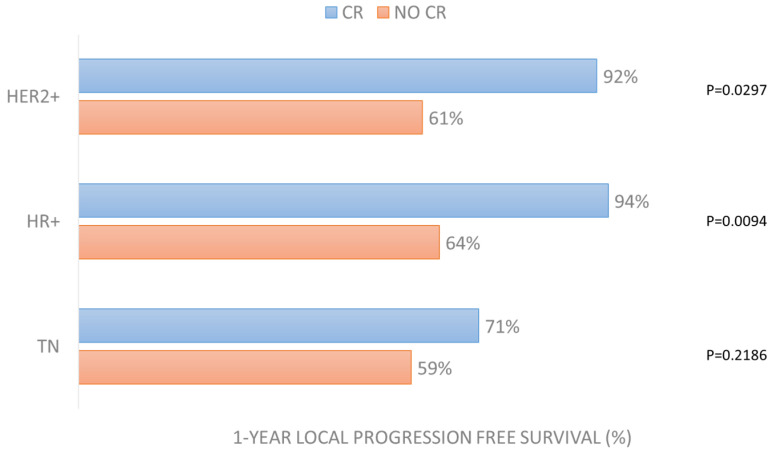
One-year local progression-free survival in the 3 groups according to CR achievement after ECT treatment.

Cox regression analysis revealed that LPFS is positively affected by the achievement of complete response to ECT treatment in the HER2+ (*p* = 0.0297) and HR+ (*p* = 0.0094) groups (Figure 5). It is also positively affected by small lesions’ size in the HR+ group (*p* = 0.0260) and by the presence of concomitant systemic treatment (*p* = 0.0289) and treatment of multiple lesions (*p* = 0.0295) in the TN group (Table S2).

### 3.5. Overall Survival

During follow-up, 72 patients (42%) died; the distribution was similar among groups (44%, 40%, and 48%, respectively, *p* = 0.1255). Death occurred after a mean period of 12 ± 13 months (median 6.6, range 0.7–53).

The overall survival curve was significantly lower for the TN group in comparison with the HER2+ group (*p* = 0.0319) whilst not significantly different from the HR+ group (*p* = 0.1227) (see Figure 3B). One-year local progression-free survival was 65% (C.I. 48–82%) in the HER2+ group, 58% (C.I. 46–71%) in the HR+ group, and 59% (C.I. 39–79%) in the TN group.

Cox regression analysis revealed that overall survival was affected by time to local progression in all groups (*p* = 0.0065 in HER2+, *p* < 0.0001 in HR+, *p* = 0.0363 in TN). It was also affected by the oligometastatic condition in the HER2+ (*p* < 0.0001) and HR+ (*p* = 0.0202) groups (Table S3).

## 4. Discussion

This is, to our knowledge, the largest cohort study on breast cancer patients treated with ECT for cutaneous metastases to date and even the longest in terms of follow-up information.

The results in terms of local response to treatment are similar to the data already published in the literature [19], where the CR rate for treated nodules ranged from 40% to 74% in cohorts larger than 20 patients, and in a recent study [7], a CR rate of 58% was obtained in cutaneous lesions in the first INSPECT data collection on breast cancer patients. In our cohort study, a CR rate of 52% in the HER2+, 55% in HR+, and 49% in TN groups was very homogeneous and in the median region of the results available in the literature.

In this series, all patients showed a similar response to ECT, regardless of their receptor status. At the current time, no studies have been conducted to specifically evaluate if there is a difference between receptor status and response to ECT, but some studies have evaluated that amongst other parameters and have come to slightly different conclusions. In 2015, Cabula et al. [10] performed a retrospective multicenter cohort analysis where they evaluated 113 patients (and 214 tumors) treated between 2010 and 2013 in 13 Italian institutions. In their analysis, tumor size was the most powerful predictor of CR, together with absence of visceral metastases, ER positivity, and low Ki-67. In 2019, Wichtowski et al. [20] found that positivity to estrogen receptor better correlates to ECT response, while in 2021, Russano et al. [16] found that negativity to estrogen and progesterone receptor and to HER2 correlates better with ECT response. These discrepancies could be explained by the fact that all these studies were conducted retrospectively with a relatively small subset of patients. Further studies are needed to fully address this controversy.

ECT has been repeatedly demonstrated to be a safe procedure [6,21,22,23] and, in this series, we confirm this finding. All toxicities were mild, and all recovered within 2 months from the procedures. ECT could be performed not only as a palliative procedure but also as an alternative treatment to surgery in the case of patients not eligible for surgery [6] or to other standard treatments, even in the case of primary tumors, where surgery could be very mutilating, as new studies are investigating [21].

Skin involvement represents a relatively common event in the metastatic pattern of BC, with up to 30% of advanced cases in different series [24,25]. Furthermore, skin metastases are continuously under the patient’s eye, causing strong psychological distress [26]. An effective local treatment is, thus, mandatory to preserve the quality of life of patients. Surgical resection and/or radiotherapy can only be offered to a limited number of patients because of multifocality or previously irradiated tissues and on lesions that have spread to a wide area. At any rate, ECT is repeatable and can even be performed in an outpatient setting. In this context, a high responsiveness, together with the relative acceptability of the treatment in terms of pain, side effects, and discomfort, was observed in elderly patients in various studies [6,27]. Furthermore, ECT has demonstrated, in a cohort of melanoma patients, that quality of life is preserved in patients achieving a complete response to local treatment [12].

We evaluated many patient, tumor, and therapy characteristics, and we found out that ECT treatment was equally effective among all our three groups, despite their differences. Patient’s age, time since diagnosis, other treatments (both previously and concomitant), and ECT characteristics (such as electrodes used or current applied) did not result in different response to treatment. The only parameter that affected CR in all groups was lesion size. Our findings confirm what has been previously described, that tumor size is one of the main parameters or the main parameter that affects CR to ECT in these tumors [7,28]. In our study, in the HR+ group, lesion size also affected LPFS (*p* = 0.0260).

As for survival, LPFS was significatively lower in the TN group vs. HER2+ (*p* = 0.0390) and HR+ (*p* = 0.0151) groups. This could be due to different tumor biology, since TN tumors are known to be more aggressive and to have a somewhat smaller choice of systemic treatments (since both antiHER2 and hormone therapies are not indicated). In the TN group, concomitant systemic treatment (*p* = 0.0289) and treatment of multiple lesions (*p* = 0.0295) were associated with a better LPFS.

What is interesting to know is that in the “more favorable” groups, LPFS was affected by CR (in HER2+ group with *p* = 0.0297 and in HR+ group with *p* = 0.0094). Similarly, overall survival was affected by time to local progression in all groups (*p* = 0.0065 in HER2+, *p* < 0.0001 in HR+, *p* = 0.0363 in TN).

Our study shows that obtaining a CR to ECT impacts LPFS (in HER2+ and HR+ groups) and that OS is impacted by time to local progression. This correlation seems to demonstrate that a better response to a locoregional treatment could produce a benefit in overall survival. It is important to note that ECT is not a systemic treatment, but in the last few years, some studies have found a correlation between CR to ECT and OS, especially if other treatments are involved [29]. This is not the first time that a locoregional treatment seems to have produced a more systemic impact (e.g., radiotherapy and abscopal effect) [30].

The role and correlation of the immune system, of the concomitant or prior therapies and ECT, are not yet well established, and more studies are needed to better understand these phenomena. Since every correlation in our study (and in many others) was linked to lesion size, it is crucial that ECT is performed on smaller lesions to improve the probability to obtain a better CR and even better LPFS and OS. A multidisciplinary approach is needed to plan a therapeutic scenario where ECT could be performed on smaller lesions, in a time where its response could be at its best.

## 5. Conclusions

This study confirms a high local response to electrochemotherapy, which is mostly correlated to lesion size. When smaller lesions are treated, better complete responses are expected, and when complete responses are achieved, this could benefit not only the patient’s physical and psychological well-being but also their local progression-free survival, and it could also improve overall survival.

Electrochemotherapy should be considered as a therapeutical option for cutaneous and subcutaneous metastases in breast cancer patients, and its application should be discussed in a multidisciplinary team in an early referral setting.

## Figures and Tables

**Table 1:** Descriptive characteristics of the patients.

		HER2+					HR+					TN					ANOVA
#Patients		43					94					34					*p* Value
		mean	st.dev.	median	min	max	mean	st.dev.	median	min	max	mean	st.dev.	median	min	max	
age	years	65.7	12.1	69.4	37.8	88.8	65.1	12.2	66.4	31.1	94.2	61	13	60	33	84	0.1350
#nodules × patient		2.8	2.2	2.0	1.0	7.0	2.5	2.1	1.0	1.0	7.0	2.8	1.8	2.0	1.0	7.0	0.7368
time since diagnosis	years	7.8	5.7	6.3	0.4	23.3	6.8	5.2	5.5	0.1	23.0	5.8	4.3	4.5	1.3	17.1	0.3651
		n	%				n	%				n	%				
histopathology	ductal	31	72%				77	82%				31	91%				0.1031
	lobular	3	7%				9	10%				1	3%				
	dutto-lobular	1	2%				2	2%				0	0%				
	other	8	19%				6	6%				2	6%				
oligometastatic	no	7	16%				40	43%				17	50%				0.0057
	yes	35	81%				53	56%				15	44%				
	unknown	1	2%				1	1%				2	6%				
concomitant sys th	yes	30	70%				52	55%				13	38%				0.3294
	no	13	30%				42	45%				21	62%				
lesions treated ect	single	21	49%				48	51%				11	32%				0.1646
	multiple	22	51%				46	49%				23	68%				

**Table 2:** Previous treatments in the analyzed groups.

HER2+			HR+			TN		
	N	%		N	%		N	%
Surgery	35	81.4%	Surgery	78	83.0%	Surgery	32	94.1%
Chemo	36	83.7%	Chemo	81	86.2%	Chemo	30	88.2%
Radio	25	58.1%	Radio	63	67.0%	Radio	29	85.3%
Endocrine	6	14.0%	Endocrine	34	26.2%	Endocrine	0	0.0%
Targeted	2	4.7%	Targeted	8	8.5%	Targeted	0	0.0%
Unknown	2	4.7%	Unknown	1	1.1%	Unknown	0	0.0%
No	0	0%	No	2	2.2%	No	0	0.0%
#treatments per patient *								
1	8	18.6%	1	10	10.6%	1	2	5.9%
2	9	21.0%	2	20	21.3%	2	6	17.6%
3	19	44.2%	3	35	37.3%	3	26	76.5%
4	4	9.3%	4	21	22.3%	4	0	0.0%
5	1	2.3%	5	5	5.3%	5	0	0.0%
Unknown	2	4.6%	Unknown	1	1.1%	Unknown	0	0.0%
No	0	0.0%	No	2	2.1%	No	0	0.0%

* amount of treatment received by a single patient (i.e., surgery + chemotherapy = 2, etc.). Each treatment (surgery, chemo, radio, endocrine, targeted) received a point of 1.

**Table 3:** Characteristics of the treated lesions and ECT parameters.

		HER2+		HR+		TN		NOVA
#Nodules		119		237		94		*p* Value
		N	%	N	%	N	%	
Localization of nodules	Chest	109	92%	211	89%	89	95%	0.3643
	Head/neck	5	4%	11	5%	0	0%	
	Abdomen	2	2%	4	2%	3	3%	
	Back	2	2%	5	2%	2	2%	
	Upper limbs	1	1%	6	3%	0	0%	
Electrode	Linear	8	7%	73	31%	7	7%	<0.0001
	Hexagonal	111	93%	164	69%	87	93%	
Current	0–1.5	43	36%	87	37%	22	23%	<0.0001
	1.5–3	51	43%	55	23%	35	37%	
	3–5	16	13%	37	16%	12	13%	
	5–7	2	2%	9	4%	10	11%	
	7–10	1	1%	18	8%	6	6%	
	>10	0	0%	21	9%	3	3%	
	Unknown	6	5%	10	4%	6	6%	
Preirradiated		54	45%	124	52%	71	76%	<0.0001
Lymphoedema		1	1%	20	8%	21	22%	<0.0001
Small nodules (≤3 cm)		90	76%	169	71%	62	66%	0.1095
Large nodules (>3 cm)		29	24%	68	29%	32	34%	

## Data Availability

The data presented in this study are available on request from the corresponding author. The data are not publicly available due to privacy.

## Supplementary Materials

The following supporting information can be downloaded at: https://www.mdpi.com/article/10.3390/cancers15123116/s1, Table S1. Factors affecting complete response to ECT treatment. Table S2. Cox regression analyses for local progression free survival in the 3 groups. Table S3. Cox regression analyses for overall survival (OS) in the 3 groups.
