# Supplementary material for: Efficacy of Electrochemotherapy in Breast Cancer Patients of Different Receptor Status: The INSPECT Experience
Source: Cancers (Basel). 2023 Jun 8;15(12):3116. doi: 10.3390/cancers15123116 (PMC10295899; doi:10.3390/cancers15123116)
Supplement: Supplementary file 1 [file cancers-15-03116-s001.zip › cancers-2399072-supplementary.pdf]

Table S1. Factors affecting complete response to ECT treatment.

		HER2+		HR+		TN	
**COVARIATES**		**CR%**	**P VALUE**	**CR%**	**P VALUE**	**CR%**	**P VALUE**
**Oligometastatic**	yes	49%	0.7823	49%	0.2664	33%	0.6782
	no	43%		38%		27%	
**Previous sys treatment**	yes	44%	0.1912	44%	0.4902	31%	0.8055
	no	71%		56%		25%	
**Concomitant sys treatment**	yes	50%	0.9156	46%	0.7493	42%	0.5706
	no	48%		43%		31%	
**Preirradiation**	yes	52%	0.4916	52%	0.0300	29%	1.0000
	no	41%		29%		29%	
**Lymphoedema**	yes	*		57%	0.3990	25%	0.7705
	no			41%		30%	
**Lesions' size**	<3cm	64%	**0.0018**	57%	**0.0039**	42%	**0.0436**
	>3cm	13%		27%		8%	
**Lesions' numerosity**	single	43%	0.4434	40%	0.3099	10%	0.0943
	multiple	55%		50%		39%	
**Electrode**	linear	60%	0.5953	48%	0.4093	0%	0.2380
	hexagonal	47%		38%		33%	
**Current**	<3 A	45%	0.7896	45%	0.5507	42%	0.1007
	>3 A	50%		38%		14%	

*only 1 patient with lymphedema in HER2+ group

Table S2. Cox regression analyses for local progression free survival in the 3 groups.

		HER2+				HR+				TN			
		1 yr LPFS	C.I.95%	HR	P value	1 yr LPFS	C.I.95%	HR	P value	1 yr LPFS	C.I.95%	HR	P value
Oligometastatic	Yes<br>No	79%<br>*	63%-96%	3.30	0.3740	80%<br>81%	67%-92%<br>62%-100%	1.37	0.5598	64%<br>51%	32%-96%<br>16%-87%	1.09	0.8963
Previous sys th	Yes<br>No	75%<br>100%	56%-93%<br>-	-	0.9989	79%<br>100%	68%-90%<br>-	1.77	0.5823	37%<br>*	6%-67%	2.63	0.2342
Concomitant sys th	Yes<br>No	81%<br>61%	64%-99%<br>11%-100%	1.58	0.5882	90%<br>79%	82%-98%<br>64%-94%	1.38	0.5225	86%<br>37%	60%-100%<br>0%-75%	**12.15**	**0.0289**
Preirradiation	Yes<br>No	81%<br>70%	62%-100%<br>37%-100%	1.32	0.7182	81%<br>91%	70%-93%<br>82%-100%	2.47	0.1181	64%<br>40%	40%-87%<br>0%-98%	1.14	0.8689
Lymphoedema	Yes<br>No	°<br>70%	53%-86%			*<br>79%	69%-90%	-	1.0000	67%<br>56%	13%-100%<br>29%-82%	1.54	0.5847
Lesion size	<3cm<br>>3cm	90%<br>56%	77%-100%<br>21%-90%	2.85	0.1714	95%<br>76%	88%-100%<br>61%-90%	3.40	**0.0260**	60%<br>63%	34%-86%<br>25%-100%	1.32	0.7118
Lesion numerosity	Single<br>Multiple	84%<br>74%	60%-100%<br>52%-97%	1.91	0.4410	82%<br>90%	69%-94%<br>81%-99%	1.10	0.8442	55%<br>75%	18%-91%<br>53%-98%	**5.20**	**0.0295**
Electrode	Linear<br>Hexagonal	80%<br>78%	45%-100%<br>61%-96%	1.41	0.7635	96%<br>76%	87%-100%<br>63%-89%	13.2	0.0700	*<br>67%	44%-89%	2.46	0.2629
Current	<3 a<br>>3 a	78%<br>80%	59%-96%<br>45%-100%	1.16	0.8576	80%<br>82%	68%-92%<br>66%-99%	1.18	0.7390	70%<br>45%	43%-97%<br>5%-85%	2.44	0.1740
Complete response	Yes<br>No	92%<br>61%	76%-100%<br>32%-91%	4.42	**0.0297**	94%<br>64%	81%-100%<br>47%-81%	5.45	**0.0094**	71%<br>59%	38%-100%<br>23%-94%	2.69	0.2186

* no data at 1 yr; ° only 1 patient with lymphoedema in HER2 positive group, test not applicable.

Table S3. Cox regression analyses for overall survival (OS) in the 3 groups.

		HER2+				HR+				TN			
		1 yr OS	C.I.95%	HR	P value	1 yr OS	C.I.95%	HR	P value	1 yr OS	C.I.95%	HR	P value
Oligometastatic	Yes<br>No	85%<br>*	71%-99%	+∞	**<0.0001**	71%<br>37%	57%-86%<br>17%-57%	2.25	**0.0202**	64%<br>46%	35%-92%<br>15%-78%	1.15	0.8032
Previous sys th	Yes<br>No	74%<br>54%	57%-91%<br>14%-93%	2.50	0.0943	58%<br>59%	45%-71%<br>23%-95%	0.81	0.6702	59%<br>#	39%-79%		
Concomitant sys th	Yes<br>No	68%<br>60%	49%-87%<br>24%-96%	1.19	0.7873	73%<br>70%	60%-86%<br>53%-87%	1.16	0.6688	73%<br>52%	46%-99%<br>17%-86%	1.35	0.6268
Preirradiation	Yes<br>No	82%<br>53%	64%-100%<br>25%-82%	2.13	0.1397	69%<br>77%	54%-84%<br>62%-92%	1.07	0.8370	65%<br>42%	42%-88%<br>0%-88%	1.81	0.4050

Lymphoedema	Yes<br>No	°<br>75%	 59%-91%			°<br>61%	 48%-74%			51%<br>62%	11%-91%<br>38%-87%	0.68	0.5366
Lesion size	<3cm<br>>3cm	64%<br>65%	42%-86%<br>37%-93%	1.01	0.9763	74%<br>66%	60%-87%<br>48%-85%	1.05	0.8749	70%<br>27%	47%-92%<br>0%-70%	3.44	0.0726
Lesion numerosity	Single<br>Multiple	61%<br>70%	36%-86%<br>47%-82%	1.28	0.5893	73%<br>70%	58%-87%<br>55%-85%	1.13	0.2021	44%<br>64%	0%-88%<br>42%-85%	1.04	0.9557
Electrode	Hexagonal<br>Linear	72%<br>60%	55%-88%<br>17%-100%	1.66	0.4305	60%<br>57%	45%-75%<br>33%-81%	1.05	0.9069	67%<br>58%	13%-100%<br>35%-81%	1.02	0.9813
Current	<3 Å<br>>3 Å	63%<br>88%	44%-83%<br>65%-100%	0.53	0.3206	54%<br>62%	38%-70%<br>42%-83%	0.75	0.4203	62%<br>53%	36%-88%<br>18%-88%	2.27	0.1689
Complete response	Yes<br>No	82%<br>56%	63%-100%<br>30%-82%	1.81	0.2082	67%<br>48%	51%-83%<br>29%-66%	1.81	0.0812	78%<br>49%	51%-100%<br>24%-75%	2.11	0.2533
Time to local progr$^+$				1.05	**0.0065**			1.09	**<0.0001**			1.09	**0.0363**

$^+$time to local progression is a continuous variable. * no data at 1 yr; ° only one patient in the group, test not applicable; #no patients in the group, test not applicabl
